# On the connection between level of education and the neural circuitry of emotion perception

**DOI:** 10.3389/fnhum.2014.00866

**Published:** 2014-10-27

**Authors:** Liliana R. Demenescu, Adrian Stan, Rudie Kortekaas, Nic J. A. van der Wee, Dick J. Veltman, André Aleman

**Affiliations:** ^1^Department of Psychiatry and Psychotherapy, University Hospital Aachen, RWTH AachenAachen, Germany; ^2^Department of Neuroscience, University Medical Center Groningen, University of GroningenGroningen, Netherlands; ^3^Clinical Affective Neuroimaging Laboratory (CANLAB), Leibniz-Institute for Neurobiology and Otto-von-Guericke-UniversityMagdeburg, Germany; ^4^Laboratoire des Solides Irradiés, École Polytechnique, PalaiseauFrance; ^5^Department of Psychiatry, University Medical Center LeidenLeiden, Netherlands; ^6^Department of Psychiatry, VU University Medical CenterAmsterdam, Netherlands; ^7^Faculty of Psychology, University of GroningenGroningen, Netherlands

**Keywords:** emotion, facial expressions, educational attainment, neural response, psychophysiological interaction

## Abstract

Through education, a social group transmits accumulated knowledge, skills, customs, and values to its members. So far, to the best of our knowledge, the association between educational attainment and neural correlates of emotion processing has been left unexplored. In a retrospective analysis of The Netherlands Study of Depression and Anxiety (NESDA) functional magnetic resonance imaging (fMRI) study, we compared two groups of fourteen healthy volunteers with intermediate and high educational attainment, matched for age and gender. The data concerned event-related fMRI of brain activation during perception of facial emotional expressions. The region of interest (ROI) analysis showed stronger right amygdala activation to facial expressions in participants with lower relative to higher educational attainment (HE). The psychophysiological interaction analysis revealed that participants with HE exhibited stronger right amygdala—right insula connectivity during perception of emotional and neutral facial expressions. This exploratory study suggests the relevance of educational attainment on the neural mechanism of facial expressions processing.

## Introduction

A large body of evidence substantiates the general consensus that individual differences, cognitive performance, and cerebral structure and function are modulated by environmental aspects such as cultural exposure, occupational attainment, and cognitive enrichment factors (Scarmeas and Stern, 2003; Ansari, 2012). Education is the formal process by which a social group transmits accumulated knowledge, skills, customs and values to its members. This process requires not only the strict transmission of information and the use of learning skills, but it also has a formative effect on the way the members of a social group think, feel and act (Bobo and Licari, 1989; Pekrun et al., 2002; Tang et al., 2006). In fact, strong evidence suggests that this formative effect occurs at the behavioral level, e.g., highly educated individuals are more likely to have a higher standard of living (Murrell et al., 2003; Wight et al., 2006), to cope better with stressful events (Horri et al., 2010), and are less likely to commit crimes (Hall et al., 1986; Dahlberg and Krug, 2006). Moreover, evidence suggests that a higher education is associated with a protective effect against cognitive impairments (Coffey et al., 1999). Low education attainment is associated with low socioeconomic status (Miech et al., 1999) and with a high risk for depression (Mezuk et al., 2008) and anxiety (Thurston et al., 2006). Additionally, antisocial disorders are more frequent among people with low educational attainment (LE; Miech et al., 1999). In view of this, it seems obvious that education may have a lasting and formative effect on the emotional life of individuals.

Whereas the effect of education on cognitive processes is well studied (Lee et al., 2003; van Hooren et al., 2007; Brayne et al., 2010), less is known about the relation between education and emotion processing. Behavioral studies have shown a positive relation between educational attainment and emotion recognition ability (Wolfgang and Cohen, 1988; Mill et al., 2009; Trauffer et al., 2013; Demenescu et al., 2014). The present study takes a step forward by investigating the relation between educational attainment and the neural mechanisms involved in perception of facial expressions.

Facial expressions are essential for social communication. They relay the emotional states and the intentions of others. At an individual level, a wide network is involved in emotional processing. This network includes fusiform area, amygdala, medial prefrontal cortex (mPFC), insula and anterior cingulate cortex (ACC; Phan et al., 2002, 2004). Amygdala is considered a key component in the neural circuit involved in emotional evaluation (Fitzgerald et al., 2006). In addition to its role in evaluation of salient stimuli (Santos et al., 2011) and coping with stress (Andolina et al., 2013), past studies have indicated its role in cognitive processes (Schaefer and Gray, 2007). Difficult situations, as well as motivationally relevant stimuli were reported to elicit an elevated amygdala response indicating its involvement in higher cognitive processes (see for review Schaefer and Gray, 2007). The insula has been associated with evaluation, experience and expression of “internally generated” emotions (Phan et al., 2004). Research in animals showed that the insula has strong anatomical connections with the amygdala (Augustine, 1996), and in humans they both have been associated with emotional evaluation and response (Phillips et al., 2003). Another key area involved in the emotional processing network is the ACC. This area has extensive connections with subcortical structures, e.g., amygdala (Amaral and Price, 1984).

Observing the above mentioned literature and considering the neural network involved in emotional processing, we investigated whether educational attainment, i.e., the level of education, modulates the neural response associated with emotional perception. To test this hypothesis we used a part of the data in The Netherlands Study of Depression and Anxiety (NESDA^1^). NESDA is a multicenter, longitudinal study investigating cognitive and emotional processing in affective and anxiety disorders (Penninx et al., 2008; Demenescu et al., 2011; van Tol et al., 2011, 2012). The present study is an exploratory one in the sense that it is not part of the original aim of the NESDA. It aims at examining the possible role of educational attainment on the neural mechanism of implicit emotion perception in healthy participants.

To the best of our knowledge, there is no previous study examining the relation between educational attainments and emotional processing at the neural level. The importance this relation is emphasized by the large number of neuroimaging studies that match their groups on educational level and, in as far as emotion recognition and assessment are concerned, from the understanding of the role played by the educational attainment in the emotional life of the individuals. We assessed differences on the neural response involved in face perception between healthy volunteers with higher educational attainment (HE) and those with intermediate-to-LE. We defined the groups of subjects with HE and with LE based on the mean of the variable “Years of education” presented in Table 1. No other reference to educational system or general population is implied by this definition.

**Table 1:** **Samples characteristics and behavioral data; mean (standard deviation)**.

	HE (***n*** = 14)	LE (***n*** = 14)
**Years of education**	16.28 (1.54)	11.57 (0.85)
**Age**	39.71 (8.52)	38.79 (9.06)
**Female (%)**	42.9	50.0
**Right handed (%)**	78.6	100
**BAI**	1.43 (1.55)	2.71 (3.24)
**MADRS**	1.36 (2.37)	0.79 (1.80)
**RT angry (>scrambled)**	74.78 (93.04)	79.62 (91.26)
**RT fearful (>scrambled)**	128.71 (88.14)	114.80 (109.61)
**RT sad (>scrambled)**	108.73 (76.49)	98.04 (98.55)
**RT happy (>scrambled)**	113.39 (83.83)	110.40 (90.71)
**RT neutral (>scrambled)**	118.81 (79.96)	159.59 (147.22)

*No significant group differences were found on these variables. Note: RT—reaction time, BAI—Beck Anxiety Inventory, MADRS—Montgomery-Åsberg Depression Rating Scale. The RT represents the mean reaction time difference between each facial expression and scramble faces*.

## Material and methods

### Participants

For this retrospective analysis, data of participants were selected from the functional magnetic resonance imaging (fMRI) part of the multicenter longitudinal cohort study NESDA. A detailed description of the fMRI NESDA study including participants and selection criteria is presented elsewhere (Demenescu et al., 2011; van Tol et al., 2011, 2012). Briefly, exclusion criteria for the healthy participants were: (1) a lifetime diagnosis of Diagnosis and Statistical Manual of Mental Disorders IV (DSM IV) axis I and/or axis II, psychotic disorder or dementia; (2) a history of head injury or seizure; (3) substances abuse; (4) heavy smokers; (5) hypertension (blood pressure > 180/130 mm Hg); (6) older than 56 years; and (7) MRI incompatibility. Before the scanning session depression and anxiety symptoms were assessed using Montgomery-Åsberg Depression Rating Scale (MADRS; Montgomery and Asberg, 1979) and Beck Anxiety Inventory (BAI; Beck et al., 1988). Participants were scanned in three main centers from Netherlands: University Medical Center of Groningen (UMCG), Academic Medical Center (AMC) Amsterdam, and Leiden University Medical Center (LUMC). All participants were native Dutch speakers.

Out of the 58 healthy participants included in the main NESDA study (Demenescu et al., 2011), we discharged two participants due to head movement >3 mm, and one because of dropout from the educational system after 5 years. The remaining participants were split in two groups as follows: 14 participants with 9 to 12 years of education were defined as the *low level of education* (LE) group. As age (Mill et al., 2009) and sex (Donges et al., 2012; Demenescu et al., 2014) were reported to influence the ability to recognize emotions, we matched on these variables. By randomly choosing participants from the remaining sample of 41 subjects, we included 14 participants in the group with a *high level of education* (HE) corresponding to 13 to 18 years of education.

### Ethics statement

The study was approved by the Ethical Review Boards of UMCG, AMC, University of Amsterdam and LUMC and was conducted in accordance with the Declaration of Helsinki. Written informed consent was obtained from each participant prior to the scan procedure.

### Task paradigm

We employed an implicit event-related design for perception of facial expressions (Wolfensberger et al., 2008; Demenescu et al., 2011). Implicit emotion processing elicits stronger amygdala response relative to explicit emotion processing (Critchley et al., 2000). Participants viewed color pictures of facial expressions depicting angry, fearful, sad, happy, and neutral faces selected from Karolinska Directed Emotion Faces System (KDEF; Lundqvist et al., 1998) and scrambled faces used as control condition. Twenty-four pictures (twelve female and twelve male actors) were selected for each of the five facial expressions. Each particular face was not presented more than four times. The scramble face was presented eighty times. In total 200 pictures were presented in a pseudo-random order, with each picture being presented for 2.5 s, and having an interstimulus (black screen) interval of 0.5 to 1.5 s. Images were projected onto a translucent screen at the end of the scanner bed, visible via a mirror placed at a distance of 11 cm from the participants’ face. The distance between the mirror and the screen was 64 cm and the images covered roughly 18 degrees of the visual field. During the presentation of facial expression participants were instructed to indicate the gender of face. To control for brain activation associated with the pressing of a button, during the showing of scrambled faces, the participants were asked to press the right- or left-hand button corresponding to respective arrows displayed on the screen. The responses and reaction times (RT) were also recorded. In the scanning session, the paradigm was presented using E-Prime software (Psychological Software Tools, Pittsburgh, PA, USA).

### MRI acquisition and analysis

Images were acquired on a Philips 3T MR-system located at the Neuroimaging Center UMCG, AMC, LUMC using a SENSE-8 channel coil at UMCG and LUMC, and SENSE-6 head channel coil at AMC. For each participant a series of echo planar imaging (EPI) volumes—sensitive to the blood oxygenation level dependent (BOLD) effect—were obtained, entailing a T2^*^-weighted gradient echo sequence (repetition time (TR) = 2300 ms, echo time (TE) = 28.0 ms (TE = 30 ms at AMC and LUMC), flip angle 90°, matrix size 64 × 64 (AMC and LUMC: 96 × 96), interleaved slice acquisition, 39 slices (35 at AMC and LUMC), 3 × 3 (AMD and LUMC: 2.29 × 2.29) mm in-plane resolution, and 3 mm slice thickness). Anatomical images included a sagittal 3D gradient-echo T1-weighted sequence (TR = 9 ms, TE = 3.5 ms, matrix size: 256 × 256, voxel size 1 × 1 × 1 mm, 170 slices).

Functional imaging data were preprocessed and analyzed using the statistical parametric mapping software package (SPM5) implemented in Matlab 6.5 (The MathWorks Inc.). A detailed description of the preprocessing was previously presented (Demenescu et al., 2011). The preprocessing included slice-time correction, realignment, coregistration of the T1 image to the mean EPI, spatial normalization of the EPI images to the standard Montreal Neurological Institute T1-template, and spatial smoothing using an 8 mm FWHM Gaussian kernel. Subject movement greater than 3 mm in any direction resulted in exclusion of the data from further analyses.

The significant hemodynamic changes for each condition were analyzed using the general linear model (GLM, Friston et al., 1994), with respect to the event-related response convoluted with a hemodynamic response function. Statistical parametric maps for each contrast of *t*-statistic were calculated on a voxel-by-voxel basis. *T*-contrasts for “angry > scrambled”, “fearful > scrambled”, “sad > scrambled”, “happy > scrambled” and “neutral > scrambled” were calculated for each subject. The contrast images obtained by fixed-effect analysis were entered into the second level analysis. In order to determine stimuli-specific regional responses for between-group differences, a random effect analysis was conducted employing repeated measures analysis with facial expressions as within subject factor, group (HE and LE) as between subjects factor, and centers as factor of nuisance. Significance threshold was set to *p* < 0.05 Family-Wise Error (FWE) corrected for multiple comparisons for the main effect. Region of interest (ROI) analyses were conducted to increase statistical sensitivity of a group effect on the brain areas involved in emotional processing with an initial threshold of *p* < 0.001. Small volume correction (SVC) with a threshold of p_FWE_ < 0.05 was applied within *a priori* defined regions: fusiform gyrus, amygdala, mPFC, ACC and insula using anatomical masks created with Wake Forest University PickAtlas (WFU pickatlas, Maldjian et al., 2003).

In an additional analysis we tested for an effect of educational attainment on the functional connectivity between amygdala—ACC, amygdala—insula, amygdala—fusiform and amygdala—mPFC. For this purpose a Psycho-Physiological Interaction (PPI) analysis (Friston et al., 1997) was employed. PPI is a method assessing task-dependent connectivity between a “seed” brain region and the rest of the brain. Individual time series of the left and right amygdala were extracted from the contrast faces vs. scrambled faces using a threshold of *p* < 0.05, uncorrected. The PPI term was calculated as the element-by-element product (interaction term) of the amygdala time series and a vector coding for the task effect (angry > scrambled, fearful > scrambled, sad > scrambled, happy > scrambled and neutral > scrambled). In the second level analysis, we included the contrast images for the PPI effect which, during each facial expression, indicated a positive functional connectivity of amygdala with the rest of the brain. Two separate repeated measurement analyses were conducted, one for the left and one for the right amygdala. Facial expressions were defined as within subject factor, and group as between subjects factor. Scan sites were defined as nuisance factors. The ROI analyses were conducted employing ACC, insula, fusiform, and mPFC masks defined with WFU PickAtlas using and initial threshold of *p* < 0.001 uncorrected and *p* < 0.05 FWE SVC for the ROI area. Outside these regions of interest the FWE correction was applied.

Analyses of demographic and behavioral data were conducted using SPSS 18. The RT to emotional facial expressions was calculated as the difference between non-scrambled and scrambled faces. Table 1 displays the mean and standard deviation of the RT. Independent two sample *t* tests were conducted testing for an effect of group on RT, age, BAI and MADRS score, whereas testing for group effect on gender and handedness was conducted using the Chi-squared test or the corresponding Fisher’s Exact test if the frequency of observed values in one cell was less than the expected one.

## Results

### Characteristics of the group

Characteristics of the samples are displayed in Table 1. No significant difference between higher and lower educational attainment of participants was found on age (*t*_(26)_ = 0.279, *p* > 0.05 (2-tailed)), MADRS score (*t*_(26)_ = 0.717, *p* > 0.05 (2-tailed)), BAI (*t*_(26)_ = 1.337, *p* > 0.05 (2-tailed)), gender (χ^2^(1)= 0.144, *p* > 0.05 (2-tailed)) or on handedness (Fisher’s Exact test *p* = 0.222). Also, no significant correlation was found between the participant’s age and years of education (*r*_(12)_ = −0.018). Finally, no group effect was found on the RT to facial expressions (*F*_(1,26)_ = 0.015, *p* = 0.904).

### Imaging data

Facial expressions (>scrambled) elicited increased bilateral fusiform gyrus (left: [−39, −48, −24], *Z* = 6.31 and right: [39, −42, −24], *Z* = 7.50), right middle occipital gyrus ([24, −90, 18], *Z* = 7.10), left amygdala ([−21, −6, −15], *Z* = 6.16), right middle frontal gyrus (BA46, [51, 30, −21], *Z* = 5.33) activation at p_FWE_ < 0.05 (whole-brain correction). Right amygdala ([18, −6, −15], *Z* = 4.70) activation to facial expressions across all participants was observed at a slightly more lenient threshold of *p* < 0.05 false discovery rate.

**Figure 1:**
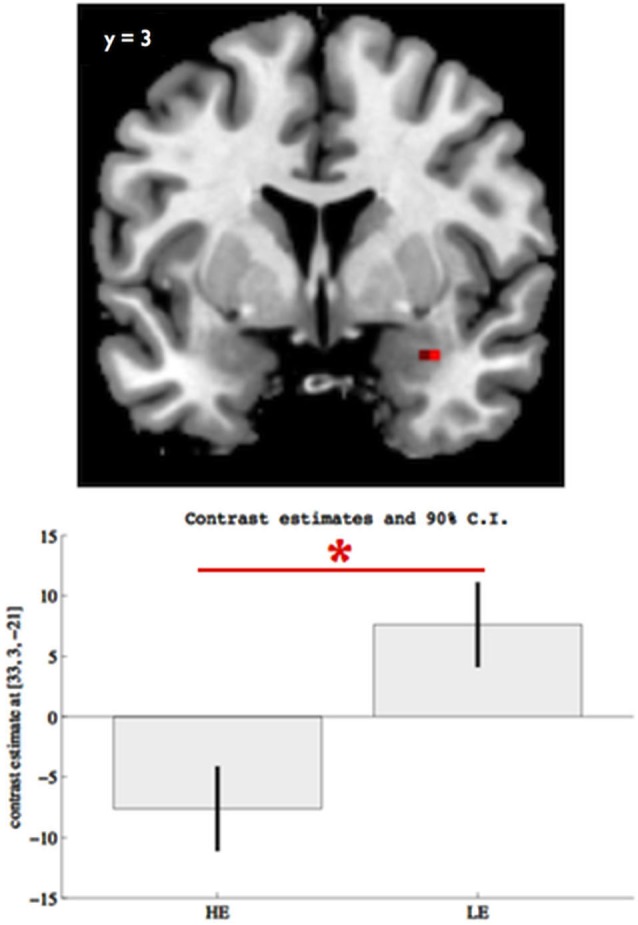
**Group differences on right amygdala (*x* = 33, *y* = 3, *z* = −21) response to facial expressions for p_FWE_ < 0.05, SVC**. The boxes indicate the amygdala mean response across all facial expressions (vs. scramble) within each group, while the whiskers indicate the 90% confidence interval. Participants with lower educational attainment (LE) showed significantly elevated right amygdala response to overall facial expression perception, relative to participants with higher educational attainment (HE). The asterisk indicates significant group differences (p_FWE_ < 0.05, SVC).

We found an effect of group in right amygdala response to facial expressions (*x* = 33, *z* = 3, *y* = −21, *Z* = 3.26, p_FWE_ = 0.024, SVC, Figure 1) with the LE participants showing a significantly higher amygdala response than the HE, when viewing facial expressions of emotion (p_FWE_ = 0.012, SVC, Figure 1). We found no other significant group effect in ACC, insula, fusiform gyrus, or mPFC, or in any other brain regions. Also, we found no significant group by faces interaction on the neural response to face perception.

We also checked that our baseline does not elicit an effect on any of the areas found activated in response to facial expressions of emotion. Thus, we conducted an independent t test, controlling for centers and, across both groups, we found a main effect of scramble face perception, in right middle occipital gyrus (*z* = 39, *y* = −87, *z* = 0; *k* = 8, *Z* = 5.32, p_FWE_ < 0.05) and no group difference.

**Figure 2:**
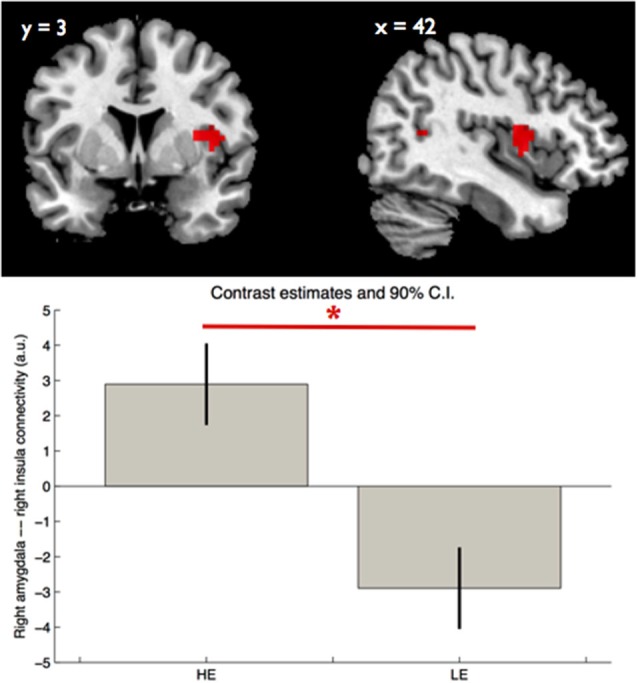
**Group main effect on right amygdala—right insula connectivity**. The boxes indicate, in arbitrary units, the strength of the right amygdala—right insula functional connectivity within each group, during perception of facial expressions, while the whiskers indicate the 90% confidence interval. The asterisk indicates significant group differences (p_FWE_ < 0.05, SVC). LE—low educational attainment and HE—high educational attainment.

Regarding amygdala functional connectivity we found an effect of group on right amygdala—right insula connectivity ([42, 3, 9], *Z* = 3.77, *p* < 0.05 FWE, SVC, *k* = 11). Participants with HE showed a stronger right amygdala—right insula ([42, 3, 9], *p* < 0.05, SVC, Figure 2) connectivity during faces (>scramble) perception. We found no significant group by facial expressions interaction on either left or right amygdala connectivity.

## Discussion

The present study investigated the possible modulatory role of educational attainment on the neural circuitry involved in processing of facial expressions. The results support the hypothesis of a putatively formational effect of education, that may affect emotion processing in general. We found that at the neural level, amygdala response to facial expressions was negatively modulated by educational attainment. Additionally, we found that the connectivity of the right amygdalar complex with insula was also associated with educational attainment in a positive manner. Our study provides—to the best of our knowledge—the first neuropsychological evidence of the modulatory association of education on perception of facial expressions.

### Amygdala group differences during faces viewing

Our findings are in line with previous studies on amygdala response to facial perception (Todorov, 2012). We found that viewing faces elicited an elevated amygdala response. We found an effect of the educational attainment on right amygdala. HE participants showed reduced right amygdala response when viewing facial expressions, while LE participants showed stronger amygdala response. The absence of a significant group by facial expressions interaction, indicates a rather general than emotion specific effect of education attainment on amygdala response. Previous studies have reported an effect of education attainment on overall performance in identifying emotion (Wolfgang and Cohen, 1988; Mill et al., 2009). Other studies reported the improvement of recognition-accuracy of fearful faces with a higher education attainment (Trauffer et al., 2013; Demenescu et al., 2014), whereas the recognition of angry faces seems to be impacted in a similar manner by a low education attainment (Trauffer et al., 2013). Previous studies reported elevated amygdala response to neutral faces in a wide range of psychiatric disorders, e.g., social anxiety (Cooney et al., 2006), bipolar disorder (Rich et al., 2006), schizophrenia (Holt et al., 2006), and chronically violent men (Pardini and Phillips, 2010). In healthy volunteers, elevated amygdala activation to emotional and neutral faces has been previously reported in a visual search paradigm suggesting amygdala involvement in “signaling salience in faces” (Santos et al., 2011). These findings indicate that amygdala is responsible for perception of salience information in faces, and that neutral faces might be ambiguous stimuli.

Morris et al. (1998) reported that amygdala response and lateralization during implicit emotion perception were related to awareness (Gläscher and Adolphs, 2003; Costafreda et al., 2008; Sergerie et al., 2008; Dyck et al., 2011) and to cognitive involvement (Baas et al., 2004; Fusar-Poli et al., 2009). Right amygdala hyperactivation was associated with rapid and autonomic evaluation of emotional stimuli (Gläscher and Adolphs, 2003; Costafreda et al., 2008; Sergerie et al., 2008; Dyck et al., 2011). Fusar-Poli et al. (2009) indicated that left amygdala is involved in cognitive, detailed emotional perception, whereas right amygdala is implied in global emotional reaction. Our study investigated implicit emotional processing and thus the effect of educational attainment in right amygdala might be related to automatic emotion processing. The present study adds to the existing literature by emphasizing the role of educational attainment on amygdala response to perception of facial expressions.

### Amygdala connectivity

Another outcome of our analyses was that, relative to LE participants, subjects with HE displayed a stronger amygdala—insula functional coupling during perception of facial expressions. As in the previous analysis, no group by facial expressions of emotion interaction was found indicating a general effect of educational attainment on amygdala connectivity. Insula is implicated in “generation of affective state in response to emotive stimuli” (Phillips et al., 2003), situating it at the core of affective feelings (Lindquist et al., 2012) and sensitive to salient stimuli (Menon and Uddin, 2010). Lesion studies have shown that insula has a broad role in recognition, processing, assignment of valence, and affective arousal, of negative and positive emotions (Berntson et al., 2011). Moreover, amygdala and insula—have extensive anatomical connections (Augustine, 1996)–which is considered a bridge between affective and cognitive processes (Craig, 2009). They are involved in “identification of the emotional significance” and emotional response at the state and behavioral level (Phillips et al., 2003). Seeley et al. (2007) found that insula and amygdala, along with the dorsal anterior cingulate, the thalamus and the ventral tegmental area, form the “salience network”. This network contributes to identifying the most subjective relevant internal and external stimuli. Taken together it might be concluded that education modulates the response of the degree of subjective salience.

It may be of interest for further studies to explore the effect of educational attainment on emotional perception intensity and accuracy, and also on cognitive processes. As the lack of additional data on socioeconomic or environmental factors is a limitation of the present study, these formative effects could be considered in future studies.

## Conclusions

The present study suggests that educational attainment modulates both the neural response and neural network involved in processing facial expressions. In particular, it shows that HE may be associated with reduced amygdala responses to facial expressions and stronger amygdala—insula coupling during face perception. As emphasized by Duarte et al. (2008), it may be that through education people develop better skills to cope with social situations and are less likely to respond in an emotional way. These results show the importance of considering the formative effect of educational attainment when investigating the neural circuitry underlying emotional and neutral face perception, as there is no evidence that such effect is limited to the perception of facial expressions of emotion investigated in this study.

## Conflict of interest statement

The authors declare that the research was conducted in the absence of any commercial or financial relationships that could be construed as a potential conflict of interest.

## Footnotes

^1^http://www.nesda.nl
